# Cancer Stem Cell Assay for the Treatment of Platinum-Resistant Recurrent Ovarian Cancer

**DOI:** 10.24966/srdt-2060/100076

**Published:** 2021-09-09

**Authors:** Candace M Howard, Stephen Bush, Nadim Bou Zgheib, Seth T Lirette, Antonio Cortese, Antonio Mollo, Jagan Valluri, Pier Paolo Claudio

**Affiliations:** 1 Department of Radiology, University of Mississippi Medical Center, Jackson, MS, USA; 2 Gynecologic Oncology, Charleston Area Medical Center Hospital, Charleston, WV, USA; 3 Gynecologic Oncology, Edwards Comprehensive Cancer Center, Joan C. Edwards School of Medicine, Marshall University, Huntington, WV, USA; 4 Department of Data Science, University of Mississippi Medical Center, Jackson, MS, USA; 5 Department of Medicine and Surgery, University of Salerno, Italy; 6 Obstetric and Gynecologic Unit, Department of Medicine and Surgery, University of Salerno, Salerno, Italy; 7 Translational Genomics Research Institute, Department of Biological Sciences, Marshall University, Huntington, WV, USA; 8 Department of BioMolecular Sciences, National Center for Natural Products Research, University of Mississippi, University, MS, USA; 9 Cancer Center & Research Institute, Departments of Radiation Oncology and Maxillofacial Surgery, University of Mississippi Medical Center, Jackson, MS, USA

**Keywords:** Cancer stem cells, Chemotherapy, ChemoID, Ovarian cancer, Personalized medicine, Platinum resistant ovarian cancer

## Abstract

**Background:**

Disease recurrence and progression of ovarian cancer is a common event, which is accompanied by the development of platinum-resistant or refractory disease. The presence of chemo-resistant Cancer Stem Cells (CSCs) contribute to tumor propagation, maintenance, and treatment resistance of this difficult to treat disease. We have developed ChemoID, a cytotoxic synergy assay against CSCs that identifies the most effective chemotherapy treatment from a panel of FDA-approved chemotherapies using fresh cancer biopsies

**Patients and methods:**

Ascites or interventional radiology biopsies were collected under physician order from 78 consecutive patients affected by 3^rd^ relapsed ovarian cancer. Test results from the assay were used when possible to treat patients with the highest cell kill drugs, taking into consideration their health status and using dose reductions, if needed. A chart analysis and review of CT and PET scans were performed to determine patients’ outcomes for tumor response, Progression-Free Survival (PFS), and Overall Survival (OS).

**Results:**

We observed that recurrent ovarian cancer patients treated with high-cell kill chemotherapy agents guided by the CSCs drug response assay had an improvement in their median PFS and OS when compared to historical median PFS and OS and/or when compared to patients who could not receive high cell kill chemotherapies (PFS low cell kill 3.5 months vs. high cell kill 12.0 months; OS low cell kill 6.0 months vs. high cell kill 15.0 months).

**Conclusion:**

This data indicates that the drug cytotoxicity assay aimed at targeting CSCs may be a useful tool for optimizing treatment selection when first-line therapy fails, and when there are multiple clinically-acceptable and -equivalent treatments available.

## 3. Introduction

Medical oncology is evolving from a conventional “one-size-fits-all” treatment strategy to a more sophisticated approach aimed at tailoringanticancer treatment with more effective drugs, to maximize the therapeutic effects for improved outcomes. Precision medicine in medical oncology is at understanding for individual patients which therapies are most effective. The majority of the approaches in precision medicine have been based on the genetic characterization of cancer [1–3]. Since the late 1990s, the basis of precision medicine has been to develop therapies able to specifically target cellular pathways involved in tumor growth and dissemination of cancer cells [2].

Several studies have been performed to discover biomarkers that predict effectiveness in chemotherapy [4]. However, modern medicine still lacks valid biomarkers that can help oncologists identify patients who will benefit from a chemotherapy regimen versus those who will instead suffer side effects from these agents.

Therefore, there is an unmet demand for the development of diagnostic assays that can direct individualized treatments for cancer patients. Assays that measure the response of tumor cells focused on various types of cancer including ovarian cancer [5–7], gastric cancer [8], colorectal adenocarcinoma [9], breast cancer [10,11], non-small cell lung cancer [12], and small-cell lung cancer [12,13]. The majority of these assays, which have been developed in the past 20–30 years, used tumor cell cultures that may also contain stromal cell contamination in the tested sample, which have in part accounted for their lack of reliable determination [14]. The presence of a contaminant of stromal cells can result in misinterpretation of test results due to an imperfect determination of the overall response because stromal and epithelial chemo-reactivity profiles greatly differ. Additionally, the majority of these tests have been set up to assess chemotherapy cytotoxicity by exposing the tumor cells *in vitro* to drug concentrations that are lower than the maximum concentration measured in plasma following a treatment, known as [C]_MAX_, making the entire procedure clinically relevant. Some of these tests have been improved over the years and are currently in use by progressive clinical oncologists for example in the therapy of refractory malignant tumors especially in the OBGYN setting where no many options are available for recurrent malignant ovarian tumors [15–17].

In recent years, there has been a renewed trend towards personalized treatment approaches and in this context, antineoplastic functional testing could be a further step in identifying the appropriate chemotherapeutics and molecular targeting agents.

A major advance in the understanding of cancer biology and disease progression has been the discovery of cancer cells with stem cell-like features, commonly referred to as cancer-initiating cells or Cancer Stem Cells (CSCs). These cells have been described to play a critical role in tumorigenesis, treatment resistance, and cancer recurrence [18,19]. The demonstration in 1994 that CSCs isolated from biopsies of patients affected by acute myeloid leukemia can recapitulate the full spectrum of malignant phenotypes in transplanted mice paved the road to better understanding why cancer recurs and spreads [20]. Since 2003 the presence of CSCs has been also identified in breast cancer [21] and other cancers including brain, lung, prostate, colon, and ovarian cancer [22].

A commonly used gold-standard to study CSCs and their tumorigenic potential in the laboratory is the injection of cancer stem cells in immune-deficient mice. Ordinarily, these studies are conducted by injecting CSCs in nude mice in a limiting dilution assay and observing after 30–45 days their tumor formation capacity confirmed by necropsy. It is a known feature that the CSCs are endowed with self-renewal ability, therapy-resistance and immune evasion [23], and may indeed switch between dormant and proliferative states [24], which strengthen their metastatic potential [25]. Cancer lethality is mainly due to the onset of the spread of the disease, metastases, and resistance to chemotherapy. It has been demonstrated that CSCs are sheltered against widely used chemotherapeutic agents utilizing different mechanisms, including increased expression of ATP-binding cassette drug transporters, increased capacity to repair DNA following damage, and activation of pathways that increase resistance to apoptosis [26].

The selection of the most effective chemotherapy is of primary importance not only when therapy is first initiated, but especially for recurrent disease. Administration of ineffective anticancer therapy is often connected to the development of more aggressive cancer cell clones that are resistant to subsequent therapies [27]. The ability to anticipate the most effective chemotherapy for a given patient may help to avoid the physical, emotional, and financial burden of ineffective therapy, thereby improving their quality of life [28,29].

Anticancer drugs have a high rate of failure and testing chemotherapies in cell culture have been used to identify which drugs are more likely to be effective against a particular tumor type. Many attempts have been made over the years to develop a functional test that can provide clinically relevant treatment information. However, this approach has been limited to testing performed only on the bulk of tumor cells derived from cancer biopsies [30,31].

We developed, a clinical laboratory assay (ChemoID^®^) that tests both CSCs and bulk of tumor cells directly derived from fresh tumor biopsies to predict the most effective chemotherapy agents’ combination to treat individual cancers [28,32–37]. Targeting of CSCs is a new paradigm in personalized anticancer treatment [38]. The assay is used to predictively test anticancer drugs’ efficacy for eradicating Cancer Stem Cells (CSCs), personalized by the use of a patient’s biopsy, which is measured in the lab, and not in the patient. The test is performed following a small tumor biopsy and the process involves growing the cancer cells and the CSCs from individual patient tumor biopsies in a medium that is unfavorable to normal stromal cells. Then those cellular populations are treated with various standard-of-care chemotherapeutic agents selected by the patient’s oncologist to determine how many tumor-derived cells and CSCs are killed using single drugs or their combinations. A response curve is generated for each drug and drug combination evaluated, and the data are presented graphically as a cytotoxic index. The test allows the optimum selection of chemotherapy drug(s) and has been designed to increase patient survival and to lower treatment costs and decrease toxicity by eliminating unnecessary chemotherapies.

## 4. Methods

### 4.1. Patients

We have conducted a review of 78 consecutive female 18 years and older, diagnosed with 3^rd^ relapsed EOC, who were prospectively treated with high cell-kill drugs as identified by the assay, according to their ability to tolerate the recommended treatment and using dose reductions, if needed. Biopsy specimens were obtained from standard-of-care therapeutic maneuvers (thoracentesis or paracentesis) to manage their symptoms after obtaining patients’ written informed consent. Radiology readers were blinded to patients information. Marshall University Institutional Review Board (IRB) has approved this research under protocol #326290. Fresh tissue biopsies with confirmed presence of malignant cells were collected from the Pathology lab and were sent for ChemoID^®^ testing assay by physician order. Computed Tomography (CT) scan images were collected at baseline before sample collection and after chemotherapy treatment with follow-up scans every 3 months. Repeated measures of drug response were not obtained, and therefore each subject’s data appears only once. Supportive care was allowed at the discretion of the treating physician. Response to treatment was measured by examination of CT scan and measurements using the RECIST 1.1 criteria.

### 4.2. CSCs assay

The CSCs assay procedure has been described previously [34–37].

### 4.3. Transplantation assay in immunodeficient animals

**Figure 1:**
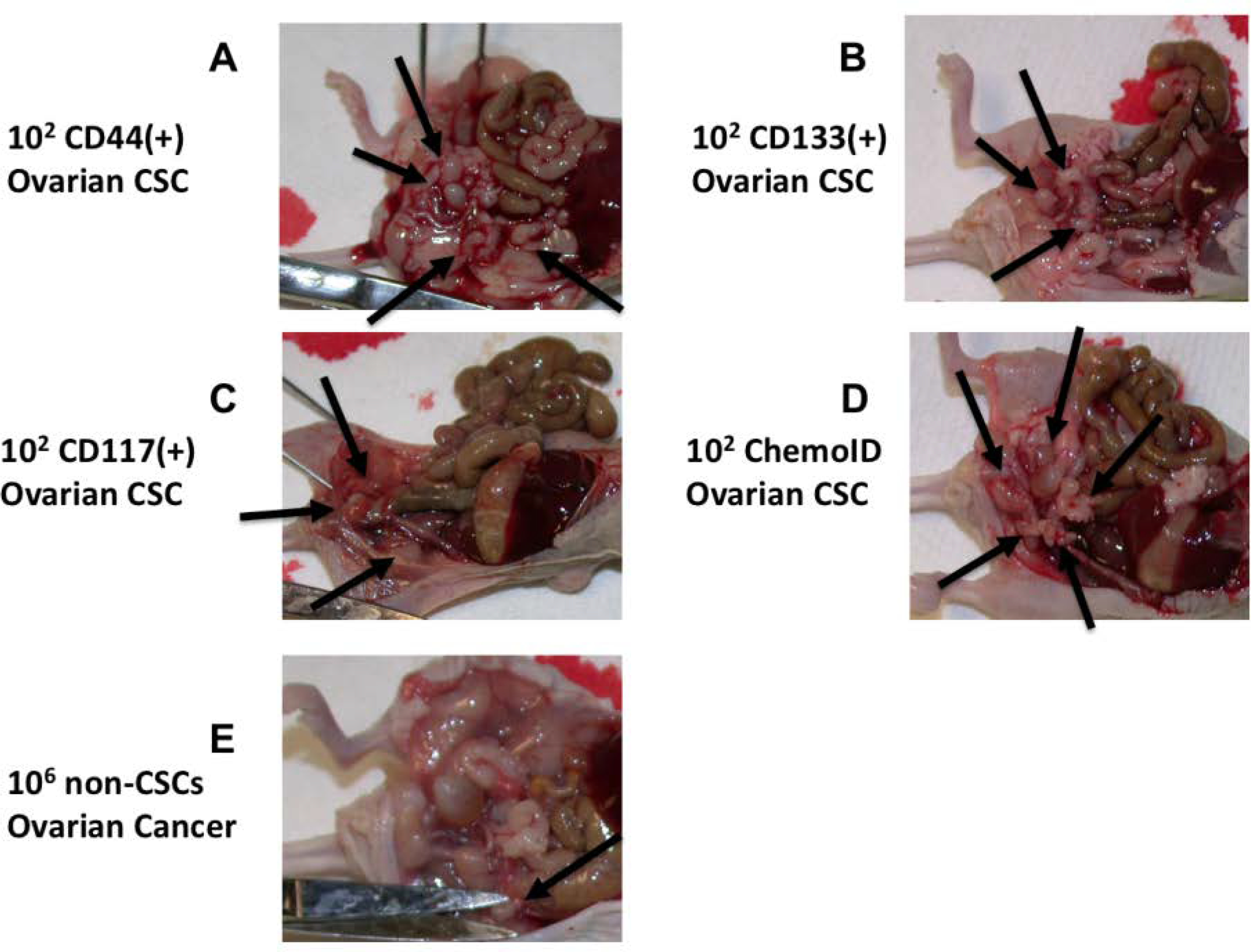
Limiting dilution assay of patient-derived CSC and non-CSC injected intraperitoneally in *nude* mice. Arrows point tumors formed following injections of CSC sorted from biopsies of ovarian cancer. **A)** Intraperitoneal tumors formed following injection of 1×10^2 CD44(+) CSCs. **B)** Intraperitoneal tumors formed following injection of 1×10^2 CD133(+) CSCs. **C)** Intraperitoneal tumors formed following injection of 1×10^2 CD117(+) CSCs. **D)** Intraperitoneal tumors formed following an injection of 1×10^2 ChemoID enriched CSCs. **E)** Single intraperitoneal tumor formed, as evidenced by the arrow, following injection of 1×10^6 non-CSCs (negative for CD44, CD133, or CD117) sorted from a biopsy obtained from a patient affected by ovarian cancer.

CSCs suspensions were injected intraperitoneally into 8-week-old female nu/nu mice (nude; Jackson Labs) (n = 6) using 1 ml syringes with a 25 G needle. Presence of tumors was evaluated by necroscopy and tumor sizes were documented by measuring the length and width of the tumors in two dimensions using a caliper. Tumor Volume (TV) was calculated using the formula volume = 1/2 × length (mm) × (width [mm])^2. Presence of tumor engraftment was recognized according to progressive nodule growth at the site of injection. Capacity to form tumors of the ovarian cancer cells was evaluated by determining presence and size of formed tumors following injection of 1 × 10^1, 1 X 10^2, 1 X 10^3, and 1 X 10^4, 1 X 10^5, and 1 X 10^6 cells in the intraperitoneal space of the nude mice and observing after 30 days their tumor formation capacity at necropsy. Figure 1 shows the tumor-initiating ability of 1 X 10^2 ovarian CSCs enriched by the ChemoID culture method compared to an equal number of CSCs sorted by amagnetic antibody and a column (Milteny Biotech, Auburn, CA) using CD44, CD117, or CD133 specific antibody following intraperitoneal injection in nude mice, compared to 1 × 10^6 non-CSC ovarian cells derived from a patient of our cohort. Column sorted and enriched CSCs were characterized by flow cytometry as previously described [33] using a C6 Accuri flow cytometer (BD Biosciences, San Jose, CA).

## 5. Statistical Analysis

Bulk of tumor responders (women who received a treatment identified by the drug response assay as 55% or above cell kill for the bulk of tumor) and CSC responders (women who received a drug in which the test identified as 40% or above cell kill of CSCs (see supplemental figures) were identified based on the cell kill of the drug used via Youden indices. Summary statistics were calculated where appropriate and all relevant graphs were constructed. Kaplan-Meier graphs were plotted and hazard ratios were calculated using Stata v.15.1 (StataCorp LP, College Station, TX). Model assumptions were graphically checked and tested via Schoenfeld residuals and were found to be satisfactory.

## 6. Results

### 6.1. ChemoID enriched CSCs cultures are tumorigenic

To demonstrate the tumorigenic capacity of ovarian CSCs enriched and prepared to be used for the ChemoID^®^ assay, we performed limiting dilution tumorigenicity experiments by injecting intraperitoneally in *nude* mice 1 × 10^2 CSCs derived from a biopsy of one of the patients affected by ovarian cancer, which were sorted by a column using specific antibodies against CD44, CD117, or CD133 (Mylteni) magnetically bound and compared their growth to 1 × 10^6 non-CSCs cells (negative for the CD markers). 1 × 10^2 CSCs enriched using the ChemoID^®^ culture system also grew abundant tumors intraperitoneally when injected in *nude* mice indicating that the tumor forming capacity of ChemoID^®^ enriched CSCs are comparable to that of CSCs sorted by using an antibody-based method. Figure 1 illustrates the tumor-forming capacity of 1 X 10^2 CSCs injected intraperitoneally in nude mice vs. 1 × 10^6 non-CSCs.

### 6.2. Real-World evidence of ChemoID^®^ assay in the personalized treatment of recurrent ovarian cancer

**Figure 2:**
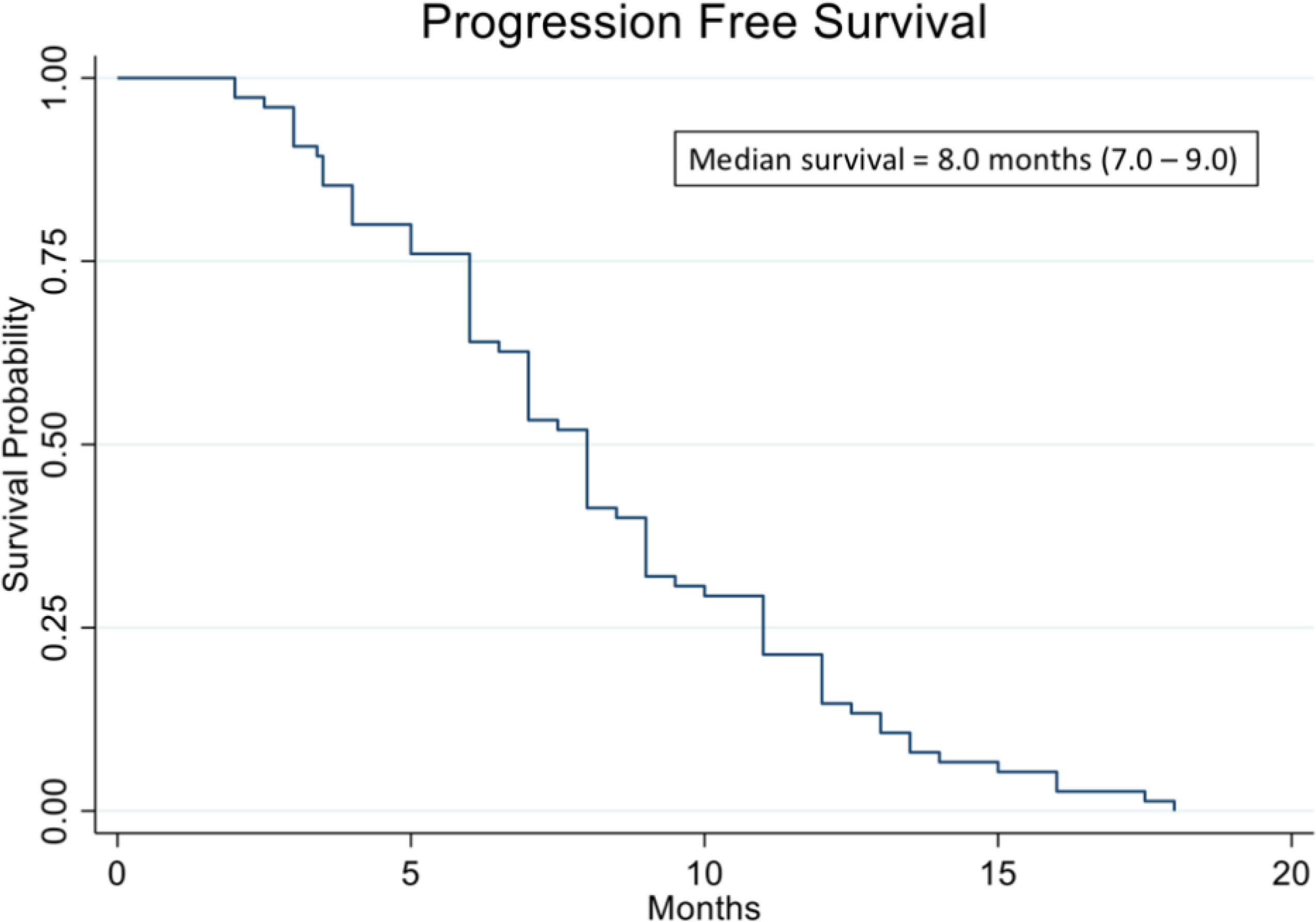
Kaplan Meier analysis of progression-free survival (PFS) of 78 real-world recurrent ovarian cancer patients treated with ChemoID-guided therapy.

**Figure 3:**
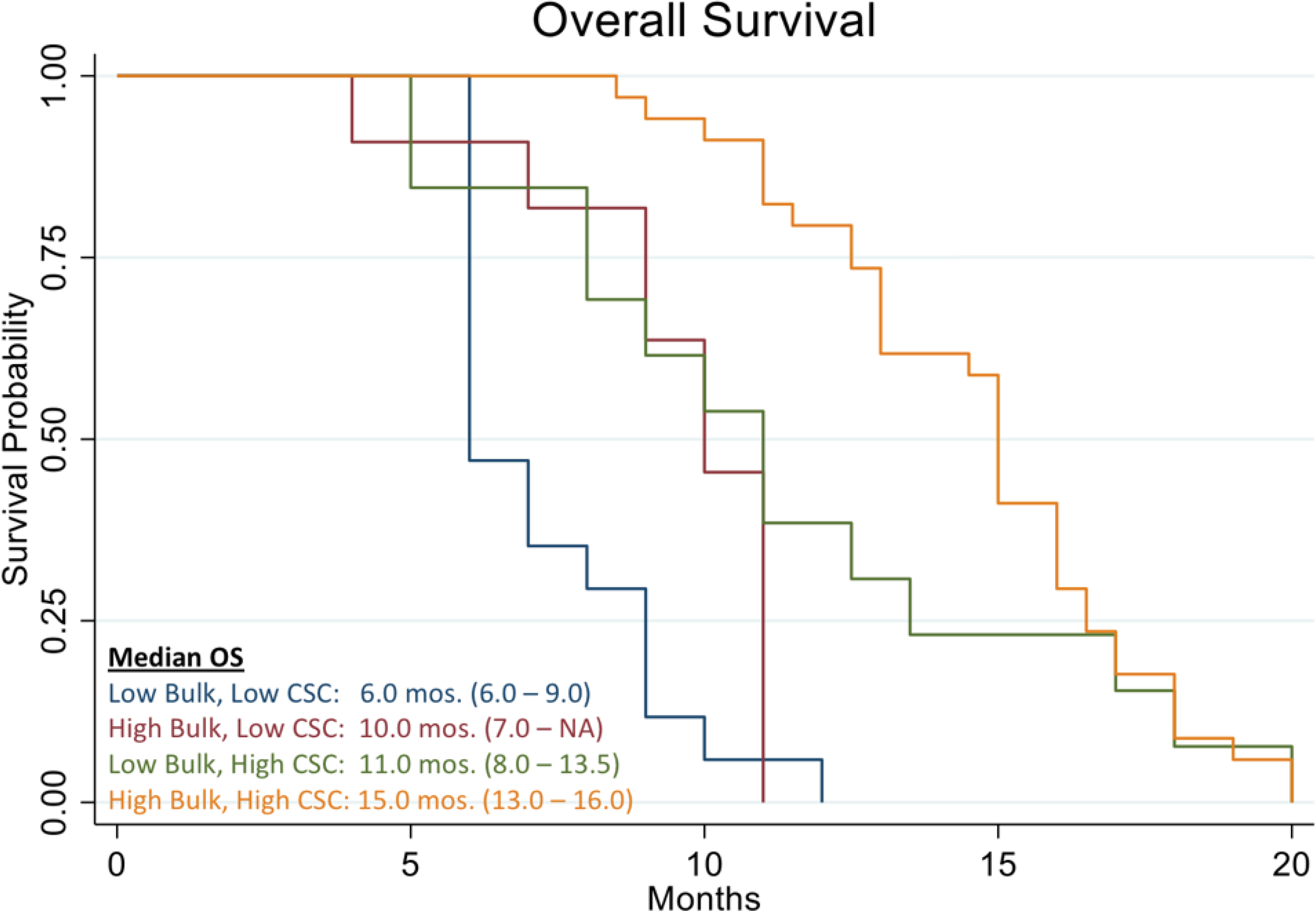
Kaplan Meier analysis of Progression-Free Survival (PFS) of 78 real-world recurrent ovarian cancer patients stratified by responding drugs vs. non responding drugs according to the percentage of cell kill on CSC and Bulk of Tumor found by the ChemoID assay.

We have analyzed data from 78 recurrent ovarian patients who were prospectively treated with cytotoxic chemotherapies guided by the ChemoID^®^ assay. Figure 2 shows the Kaplan Meier curve of progression-free survival of all patients in the study. The analysis evidenced a median PFS of 8.0 months vs. 5.6 months of historically published data [39]. Additionally, the analysis showed that patients who were treated with high cell kill chemotherapies had a median PFS of 12 months vs. that of patients who had to be treated with low cell kill drugs because of their health condition and had a median PFS of 3.5 months (Figure 3). The Hazard Ratio (HR) of progression observed between patients treated with high bulk/high CSCs cell kill drugs vs. low bulk/low CSCs cell kill drugs was 0.22 and it was statistically supported by a p-value <0.001 (Table 1). The median PFS of patients was also stratified by the cell kill of the bulk of tumor cells and CSCs based on the ChemoID^®^ assay and results are shown in table 2.

**Figure 4:**
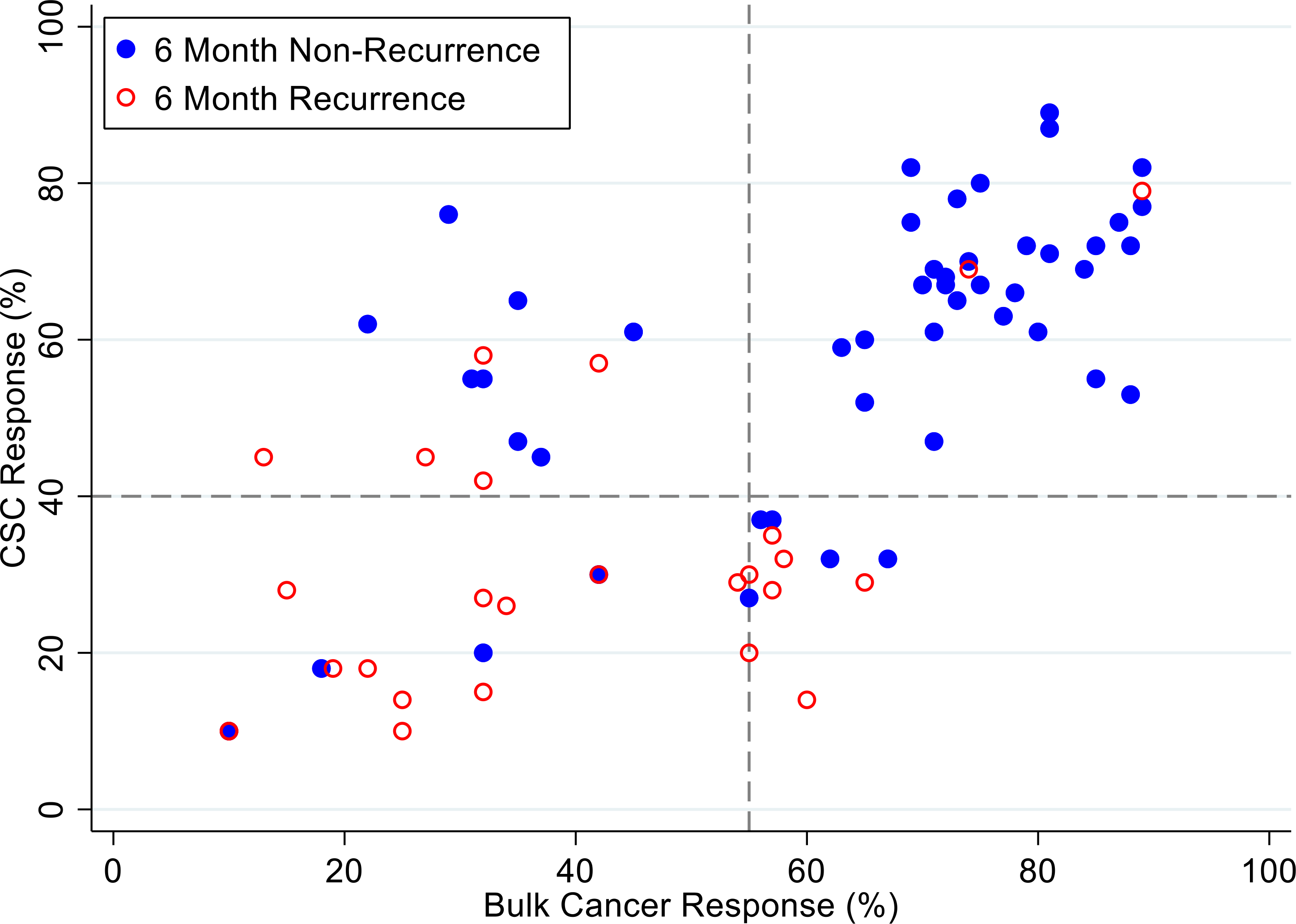
Patients are represented as red empty circles who manifested a recurrence of their ovarian cancer within 6-months from therapy start or as blue circles who didn’t manifest recurrence in the same period of time. % cell kill cut-offs were identified for CSCs (40%) and bulk of tumor (55%).

**Figure 5:**
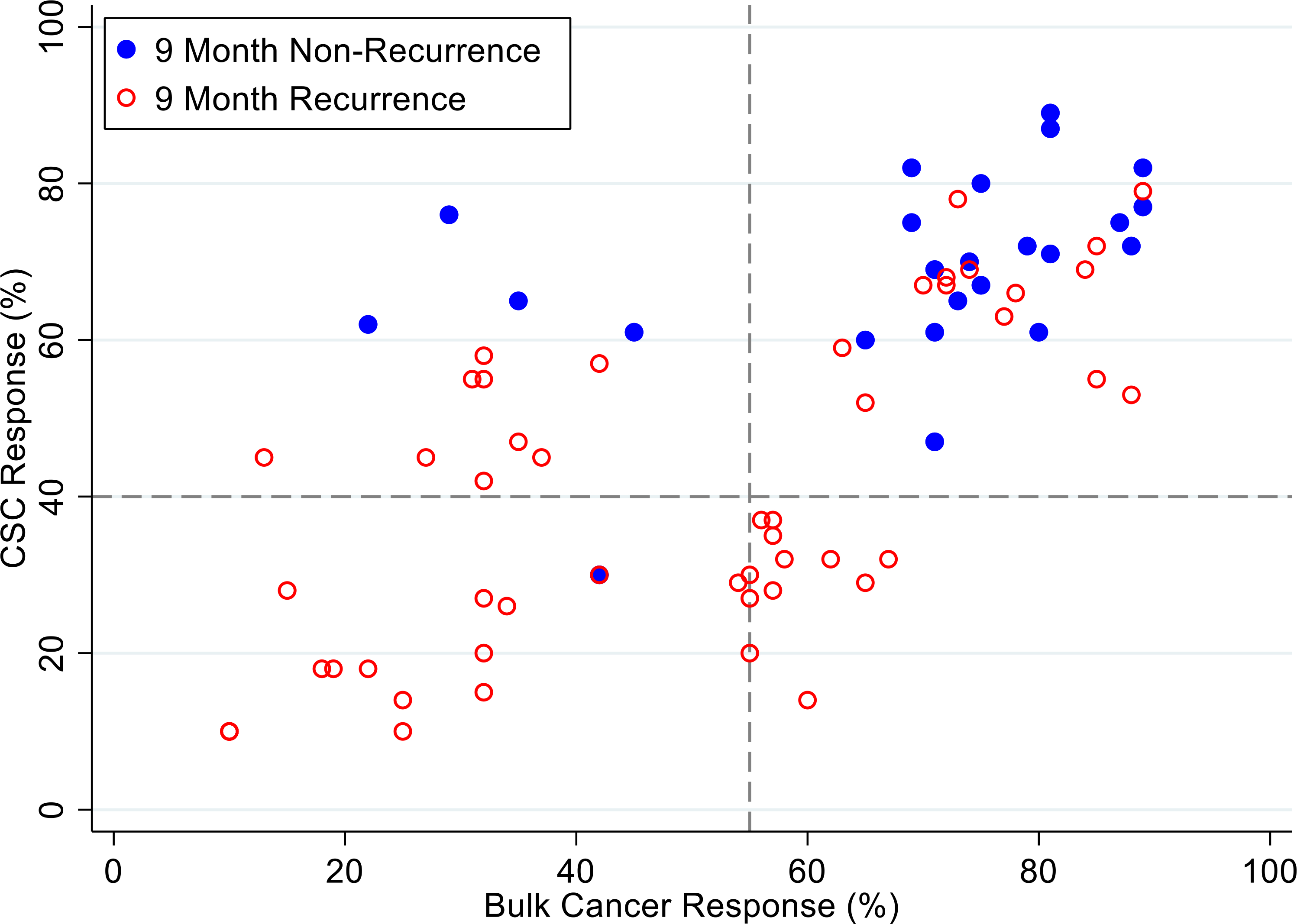
Patients are represented as red empty circles who manifested a recurrence of their ovarian cancer within 9-months from therapy start or as blue circles who didn’t manifest recurrence in the same period of time. % cell kill cut-offs were identified for CSCs (40%) and bulk of tumor (55%).

**Figure 6:**
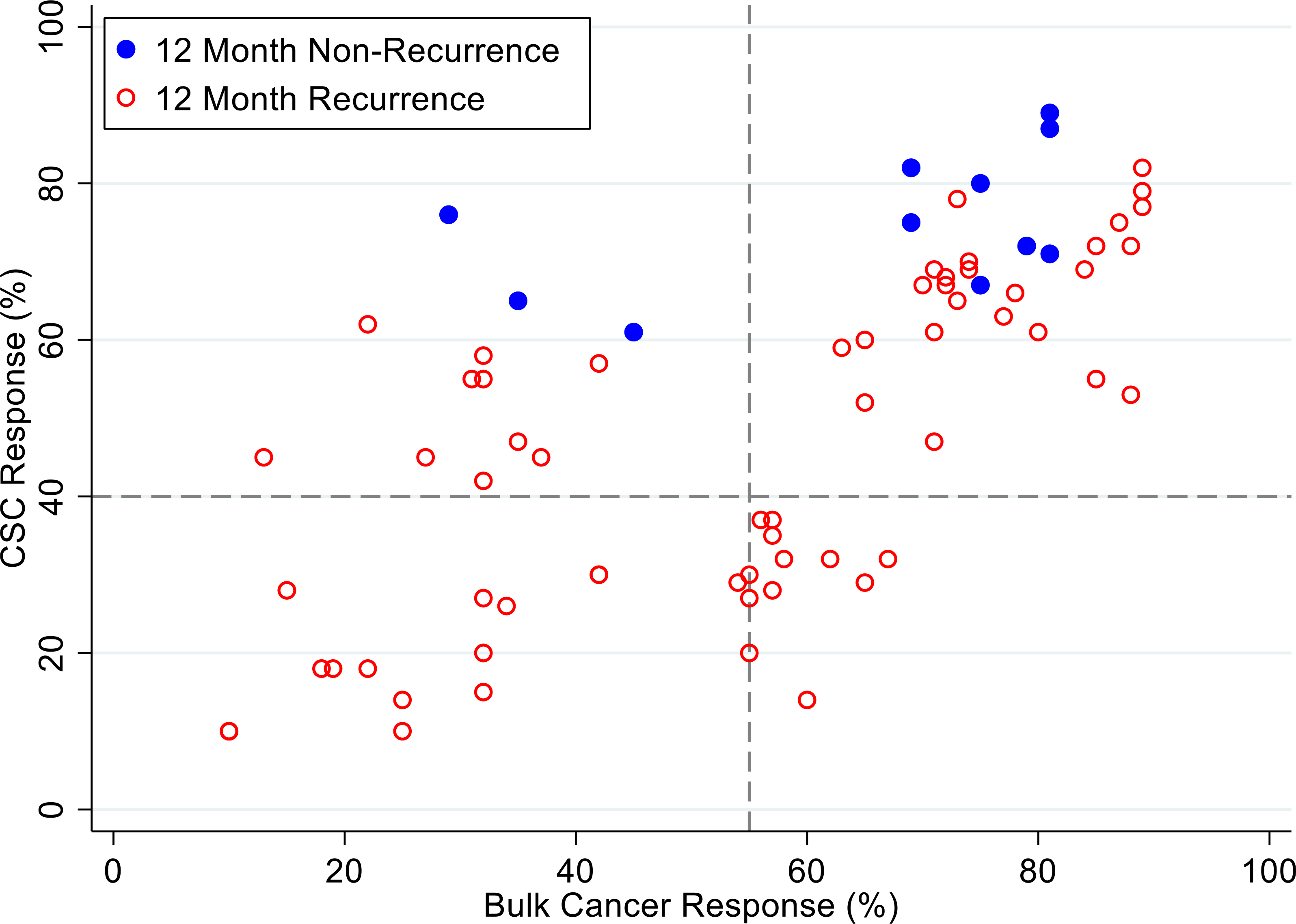
Patients are represented as red empty circles who manifested a recurrence of their ovarian cancer within 12-months from therapy start or as blue circles who didn’t manifest recurrence in the same period of time. % cell kill cut-offs were identified for CSCs (40%) and bulk of tumor (55%).

Figures 4, 5 and 6 illustrate illustrate the relationship between the CSC assay results (%-cell kill on the y-axis) and bulk tumor assay results (%-cell kill on the x-axis) characterized by 6, 9 and 12 month recurrence outcomes with solid circles representing treatment responders (patients who did not manifest a recurrence within 6–12 months from treatment) and open circles representing patients manifesting recurrence within 6–12 months from treatment. Referent lines are drawn at the optimal thresholds from the logistic regression models (40% for CSC, 55% for bulk tumor). In the upper-right quadrant are patients with high cell kill for both CSC and bulk tumor assays. In the lower-left quadrant are patients with low cell kill for both CSC and bulk tumor assays.

**Figure 7:**
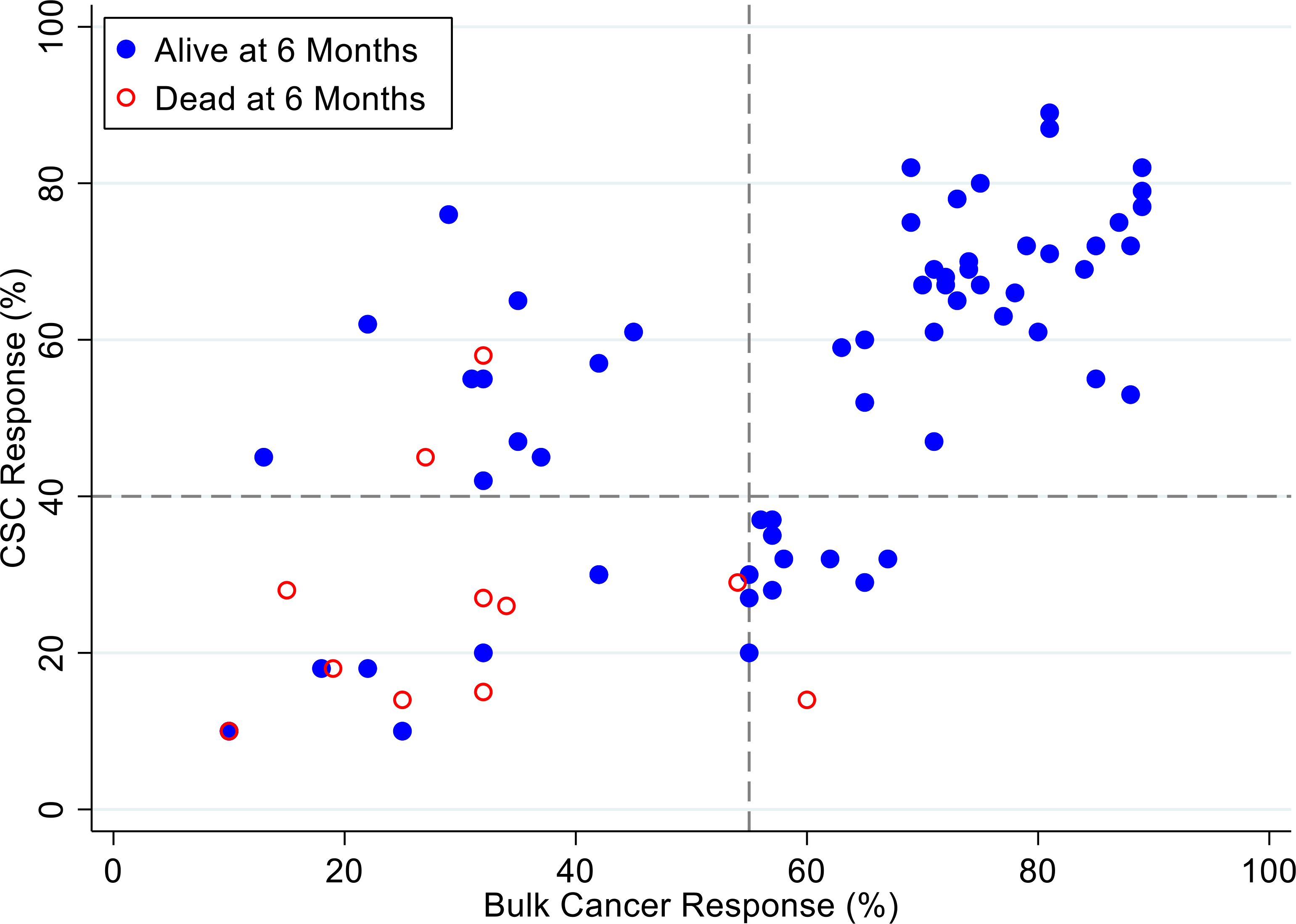
Patients represented as red empty circles died for recurence of their ovarian cancer within 6-months from therapy start. Patients represented as blue circles were alive at 6-months from therapy start. % cell kill cut-offs were identified for CSCs (40%) and bulk of tumor (55%).

**Figure 8:**
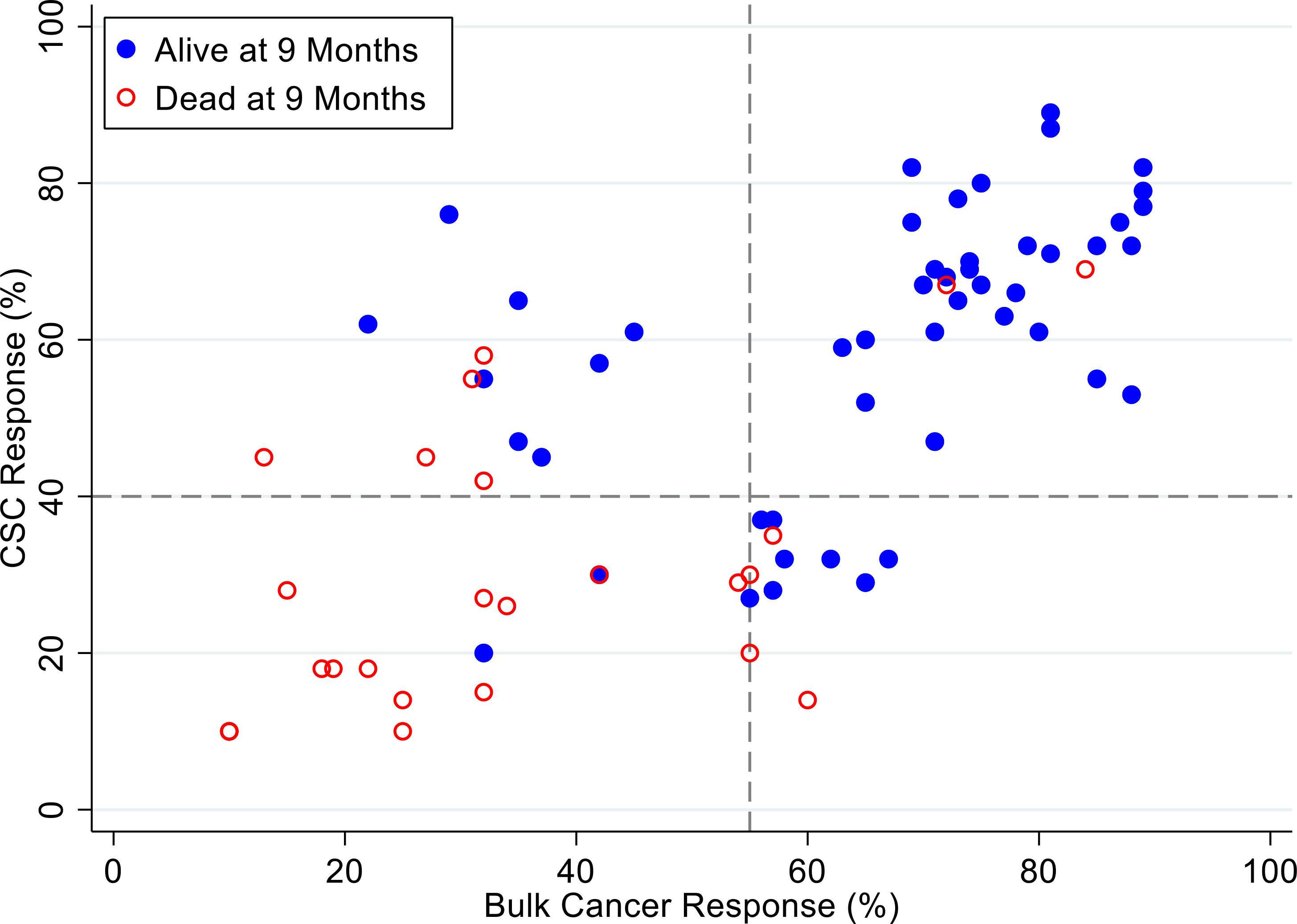
Patients represented as red empty circles died for recurrence of their ovarian cancer within 9-months from therapy start. Patients represented as blue circles were alive at 9-months from therapy start. % cell kill cut-offs were identified for CSCs (40%) and bulk of tumor (55%).

**Figure 9:**
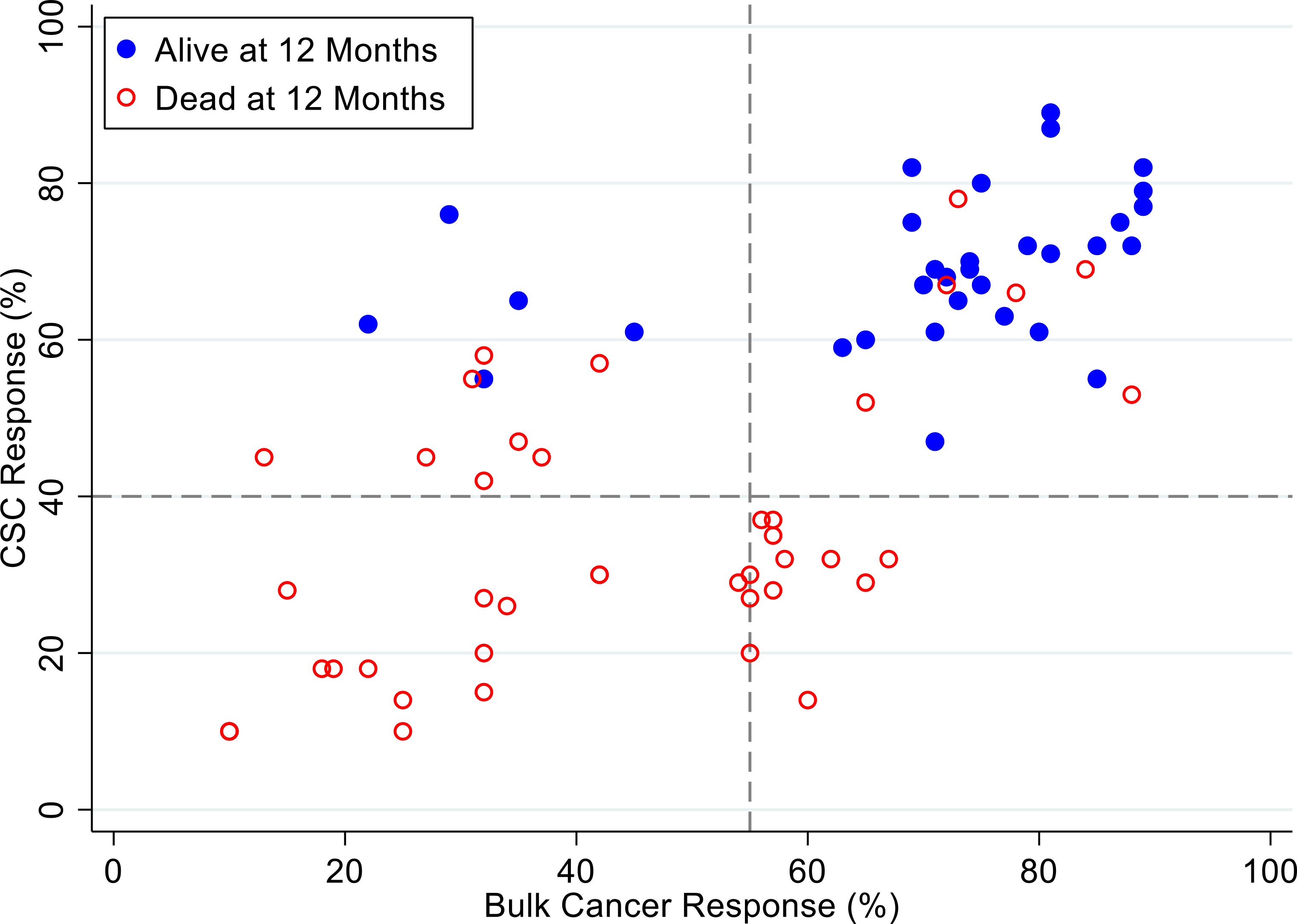
Patients represented as red empty circles died for recurrence of their ovarian cancer within 12-months from therapy start. Patients represented as blue circles were alive at 12-months from therapy start. % cell kill cut-offs were identified for CSCs (40%) and bulk of tumor (55%).

Figures 7, 8 and 9 illustrate the relationship between the CSC assay results (%-cell kill on the y-axis) and bulk tumor assay results (%-cell kill on the x-axis) characterized by 6, 9 and 12 months month survival outcomes with solid circles representing treatment responders (patients who were alive at 6, 9 and 12 months from treatment) and open circles representing deceased patients at 6, 9, 12 months from treatment. Referent lines are drawn at the optimal thresholds from the logistic regression models (40% for CSC, 55% for bulk tumor). In the upper-right quadrant are patients with high cell kill for both CSCs and bulk tumor assays. In the lower-left quadrant are patients with low cell kill for both CSC and bulk tumor assays.

**Figure 10:**
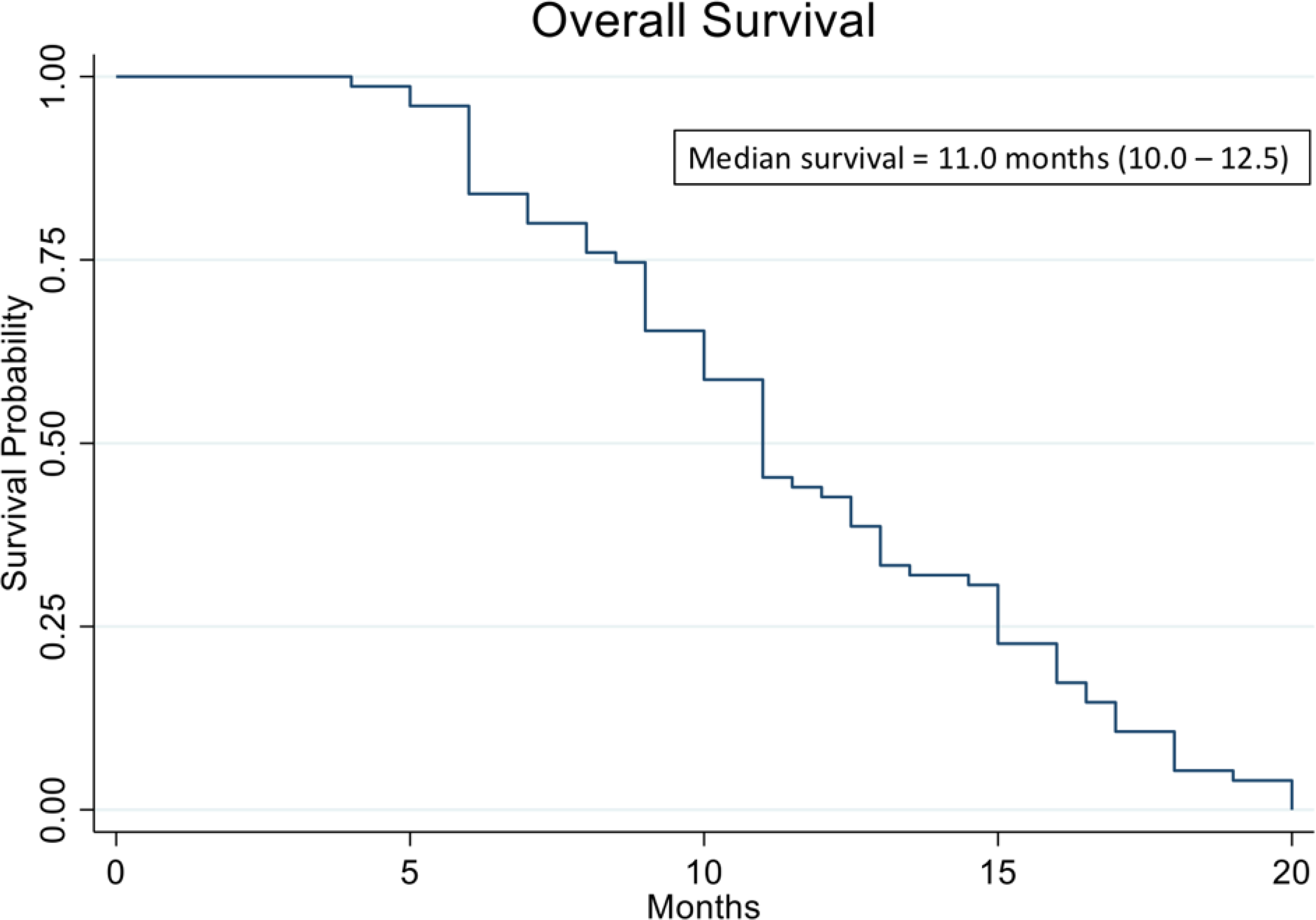
Kaplan Meier analysis of Overall Survival (OS) of 78 real-world recurrent ovarian cancer patients treated with ChemoID–guided therapy.

**Figure 11:**
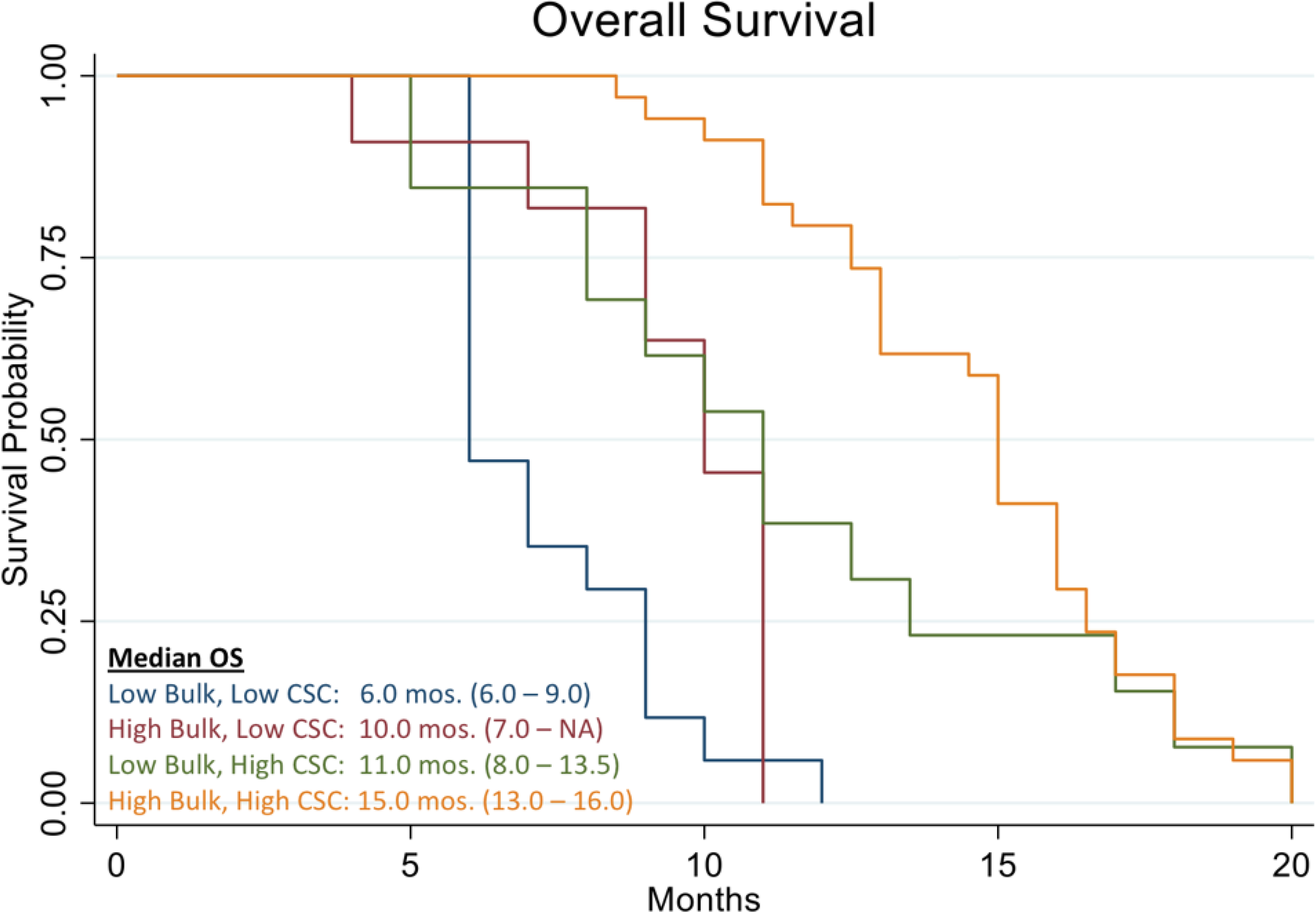
Kaplan Meier analysis of Overall Survival (OS) of 78 real-world recurrent ovarian cancer patients stratified by responding drugs vs. non responding drugs according to the percentage of cell kill on CSC and Bulk of Tumor found by the ChemoID assay.

Figure 10 shows the Kaplan Meier curve of overall survival of all patients in the study. The analysis evidenced a median OS of 11.0 months vs. 8.9 months reported in historical data for similar patients [39]. The analysis also showed that patients who were treated with high cell kill chemotherapies had a median OS of 15.0 months vs. that of patients who had to be treated with low cell kill drugs because of their health condition and had a median OS of 6.0 month (Figure 11). The hazard ratio (HR) of dying from the disease observed between patients treated with high bulk/high CSC cell kill drugs vs. low bulk/low CSCs cell kill drugs was 0.11 and it was statistically supported by a p-value <0.001 (Table 1 and Figure 11). Table 2 shows the median OS of patients stratified by the cell kill of the bulk of tumor cells and CSCs found by the ChemoID^®^ assay.

## 7. Discussion

In this work, we discussed the utility of a CSCs assay for the management of poor prognosis recurrent ovarian cancer patients. The data clearly demonstrates that recurrent ovarian cancer patients treated with high cell kill chemotherapies for bulk of tumor and CSCs had a prolonged response as determined by a shift to the right of the Kaplan Meier curve of progression-free survival and overall survival (Figures 3 and 11).

Our study assessed the association of CSCs and bulk of tumor cells assay results of recurrent EOC patients to treatment outcomes independently of other biomarkers. Patients were treated when possible with a chemotherapy regimen that was chosen among those with the highest cell kill as guided by the CSCs assay using dose reductions if needed and considering their health status. CT scans were used to monitor patients for tumor response, Progression-Free Survival (PFS), and Overall Survival (OS).

Recurrent EOC is linked with high mortality rates and a median survival of only 12–24 months that becomes increasingly worse with each additional tumor replace [39]. Regimens to treat recurrent EOC are ordinarily educated by responses to first-line therapies; therefore, the choice of which agent to use following a recurrence is usually based on toxicity profile, the previous toxicities experienced by the patient, and patient preference [3].

Aggressiveness of recurrent EOC is mostly due to the presence of Cancer Stem Cells (CSCs), which are resistant to chemotherapy and accountable for the relapse of cancer [40–43].

In a previous prospective clinical investigation, we observed that recurrent ovarian cancer patients treated with high-cell kill chemotherapy agents guided by the CSCs assay had an improvement in the median PFS corresponding to 5.4 months (3^rd^ relapse), 3.6 months (4^th^ relapse), and 3.9 months (5^th^ relapse) when compared to historical data [37]. Additionally, in our previous study we also observed that ovarian cancer patients identified as non-responders by the CSC drug response assay had 30 times the hazard of death compared to those women that were identified as responders with respective median survivals of 6 months vs. 13 months [37], which is in agreement with our current findings.

Nearly 10 different platinum-based therapies are suggestedto treat recurrent ovarian cancer patients following more than 6 months from first-line treatment (platinum-sensitive recurrent disease), and more than 20 different therapies (mostly single agents) for treatment of patients who recurr within 6 months following first-line treatment (platinum-resistant recurrent disease) [44] and treatment choices are made empirically [45].

Recent clinical trials, many of which have been supported by the Gynecologic Oncology Group (GOG), have investigated chemotherapy drugs, regimens, and reductive surgery methods in search of effective strategies to prevent EOC recurrence [37]. The AURELIA, OCEANS, and GOG-0213 phase-3 randomized trials have evaluated the use of bevacizumab in combination with chemotherapy for recurrent platinum-resistant and –sensitive ovarian cancer [46–49] and discovered that the PFS of patients treated with bevacizumab/chemotherapy was significantly prolonged (6.8 months versus 3.4 months, p<0.001) compared to patients treated with chemotherapy alone [37]. The median overall survival of these patients was 16.6 months for the bevacizumab/chemotherapy combination vs. 13.3 months for chemotherapy alone; however, this was not statistically significant (p=0.174).

Importantly none of these clinical studies focused on the idea of reducing the CSCsload in recurrent EOC to allow a longer-lasting response to therapy.

Alternative strategies should be explored given the poor response of platinum-resistant and recurrent ovarian cancer patients.

In our study, the ChemoID^®^ CSCs assay identified high-suppression drugs that contributed to a prolonged clinical response in a statistically significant manner. The current real world data analysis revealed that subjects who were treated with an assay-sensitive regimen against CSCs had an improvement in their PSF and OS compared to patients who could only receive assay-resistant regimens.

Since the toxicity is extensive for most chemotherapy drugs, it is essential to customize chemotherapy regimens based on patients’ own tumor profiles and to identify those treatments that may lead to an improvement of their PFS and OS. Based on ChemoID^®^ CSCs, we are currently conducting multi-institutional prospective clinical trial (NCT03949283) on the use of the ChemoID^®^ CSCs assay for guiding chemotherapy selection to further demonstrate the clinical utility of this test.

The ability to personalize therapy by providing the treating physician with drug response information on a panel of approved drugs should aid in the selection of the most effective chemotherapy for individual patients, thus resulting in improved clinical outcomes and lowered health care costs.

## 8. Conclusion

In conclusion, our real-world patient data indicates that the prediction of response to high cell-kill therapy against CSCs is consistent with expected improved patients’ response as measured by their PFS and OS. Reducing the CSC loads from the recurrent ovarian cancer increased the probability of OS and PFS of patients treated with assay-sensitive chemotherapies against CSCs. Our data suggest that the ChemoID CSCs assay has the potential to provide physicians with additional diagnostic information to personalize treatment with the most effective course of chemotherapy for each patient to improve outcomes of recurrent cancer.

## Figures and Tables

**Table 1:** Hazard ratio (HR) for PFS and OS of patients stratified by the cell kill of the bulk of tumor cells and CSCs found by the assay.

	PFS HR vs. Low Bulk, Low CSC	OS HR vs. Low Bulk, Low CSC
High Bulk, Low CSC	0.91 (0.25 – 3.34) p=0.885	0.83 (0.23 – 3.08) p=0.784
Low Bulk, High CSC	0.33 (0.13 – 0.85) p=0.022	0.14 (0.04 – 0.43) p=0.001
High Bulk, High CSC	0.22 (0.11 – 0.47) p<0.001	0.11 (0.04 – 0.28) p<0.001

**Table 2:** Median PFS and OS of recurrent ovarian cancer patients based upon cell kill of chemotherapies as determined by the CSCs assay.

ChemoID Cell kill	Median Age (years)	Historical PFS (months)	ChemoID Median PFS (months)	Historical Median OS (months)	ChemoID Median OS (months)
Low Bulk / Low CSC	60.5		3.5		6
High Bulk / Low CSC	65		6		10
Low Bulk / High CSC	55.5		7		11
High Bulk / High CSC	66		12		15
Overall cases	61	5.6	8	8.9	11
